# Stromal PDGFRβ Expression in Prostate Tumors and Non-Malignant Prostate Tissue Predicts Prostate Cancer Survival

**DOI:** 10.1371/journal.pone.0010747

**Published:** 2010-05-20

**Authors:** Christina Hägglöf, Peter Hammarsten, Andreas Josefsson, Pär Stattin, Janna Paulsson, Anders Bergh, Arne Östman

**Affiliations:** 1 Department of Oncology-Pathology, Karolinska Institutet, Stockholm, Sweden; 2 Department of Medical Biosciences, Pathology, Umeå University, Umeå, Sweden; 3 Departments of Surgery and Perioperative Sciences, Urology and Andrology, Umeå University, Umeå, Sweden; Uppsala University, Sweden

## Abstract

**Background:**

The identification of new prognostic markers for prostate cancer is highly warranted, since it is difficult to identify patients requiring curative treatment. Data from both experimental models and clinical samples have identified important functions of PDGFRβ on pericytes and fibroblasts in the tumor stroma.

**Methodology/Principal Findings:**

In this study the prognostic significance of PDGFRβ in prostate cancer stroma, and in matched non-malignant tissue, was evaluated with immunohistochemistry. PDGFRβ expression was analyzed in normal and tumor stroma from more than 300 prostate cancer patients. High PDGFRβ expression in tumor stroma was associated with large tumor size, advanced stage, high Gleason score and high vessel density. Perivascular PDGFRβ staining in tumors was also correlated with high Gleason score. Correlations were also observed between PDGFRβ status in tumor stroma and non-malignant stroma. Similarly, high PDGFRβ expression in adjacent non-malignant tissue stroma correlated with large tumor size, advanced stage, high Gleason score and proliferation in non-malignant epithelium. Interestingly, high levels of PDGFRβ in the stroma of tumor and non-malignant tissue were associated with shorter cancer specific survival in prostate cancer patients.

**Conclusions/Significance:**

The study revealed a number of novel associations between stromal PDGFRβ expression in prostate tumors and several important clinical characteristics, including survival.

## Introduction

Tumor behavior is, in general, largely governed by characteristics of the tumor stroma. In the case of prostate cancer this is well illustrated by a series of recent studies that have identified stromal characteristics and markers with prognostic and response-predicative significance.

Tumors with the most pronounced alteration in stroma morphology, characterized by a major loss of smooth muscle cells and increases in fibroblasts, myofibroblasts, and collagen fibres (reactive stroma grade 3), have a poor outcome compared to those with a stroma morphologically more similar to that in normal prostate tissue, i.e. reactive stroma grades 1 and 2 [1]. Recent studies have also shown that prostate cancers are characterized by loss of androgen receptors (AR) in the stroma [2]. A low frequency of stromal AR was coupled to increased Gleason score, metastasis, poor response to castration therapy and an unfavorable outcome. Furthermore, stromal characteristics, such as angiogenesis, accumulation of macrophages, lymphocytes and mast cells as well as changes in the extracellular matrix have been linked to variations in prostate tumor behaviour [3], [4], [5], [6], [7]. Notably, it has also been observed that cancer field effects and/or adaptive changes may change the stroma and glandular epithelium in the surrounding non-malignant tissue in a prognostically significant manner [2], [8], [9], [10].

PDGF α- and β-tyrosine kinase receptors exert important control functions in mesenchymal cells, such as pericytes, fibroblasts and vascular smooth muscle cells during development [11], [12]. Experimental studies have identified different functional effects of stromal PDGF receptor signaling in various tumor models. It has been demonstrated that paracrine activation of PDGF receptors on fibroblasts acts as a potent signal for tumor stroma recruitment [13], [14], [15]. Other studies have demonstrated therapeutic benefits of targeting stromal PDGF receptors involving either direct anti-tumoral effects, as well as beneficial effects on tumor drug uptake [16], [17], [18], [19], [20]. Clinical significance of the findings from experimental models is indicated by numerous studies demonstrating stromal PDGF receptor expression in different human solid tumors [13]. Most recently these studies have been supplemented with analyses revealing that high stromal PDGF receptor expression is a marker of an unfavorable outcome in breast cancer patients [21].

The biological effects of PDGF receptors in tumor fibroblasts and pericytes together with the advent of drugs with PDGF receptor-inhibitory activity, such as imatinib, sorafenib and sunitinib, thus motivates a systemic characterization of the expression pattern of PDGF receptors in human solid tumors. To this end, this study describes the expression of PDGFRβ in approximately 300 cases of prostate cancer and in matched surrounding non-malignant prostate tissue, and also reports on associations between PDGFRβ expression and molecular, histopathological and clinical characteristics.

## Results

### Variable PDGFRβ expression in normal and tumor prostate tissue

In order to evaluate the significance of PDGFRβ in prostate cancer, a TMA containing matched non-malignant and tumor tissue from 377 prostate cancer patients with up to 25 year of follow- up was analyzed by PDGFRβ immunohistochemistry.

**Figure 1:**
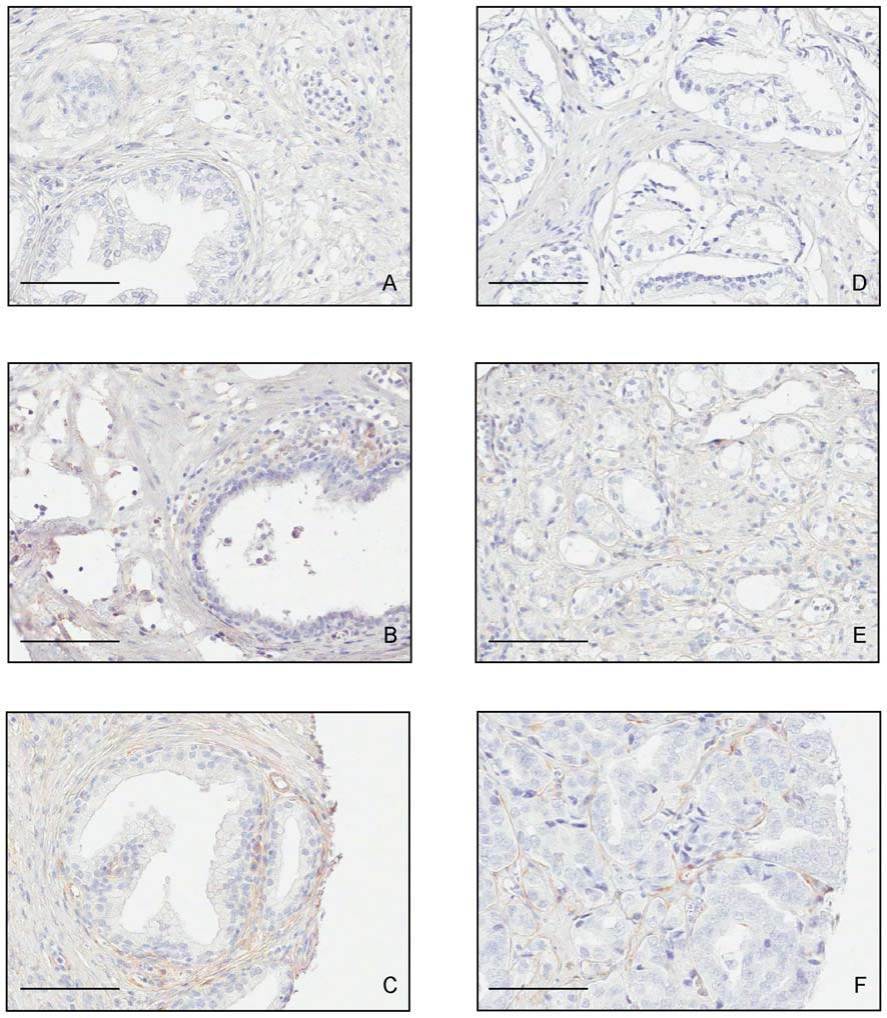
PDGFRβ expression varies between prostate cancer patients in non-malignant and tumor tissue. Sections stained for the PDGFRβ from non-malignant (A, B and C) and tumor (D, E and F) tissue from prostate cancer patients. Cases A and D show negative staining, B and E show moderate stromal staining whereas C and F present a strong stromal staining. Scale bar = 200 µM.

In agreement with previous studies, PDGFRβ expression was predominantly found in the fibromuscular stroma and in perivascular cells (Figure 1 and Table 1). In the non-malignant prostate 27% and 18% were scored as having positive PDGFRβ expression in the fibromuscular stroma and perivascular cells, respectively. Perivascular and stroma staining were not correlated. In the tumor areas 34% were positive in the fibroblast-like stroma and 17% in the perivascular cells. Staining in tumor stroma and around tumor blood vessels were correlated (Rs = 0.59). Significant associations were also observed between the PDGFRβ status in the malignant and non-malignant stroma (Table 1).

**Table 1:** Bivariate correlations.

		Non-malignant stroma PDGFRβ IR	Tumor stroma PDGFRβ IR
Tumor stroma PDGFRβ IR^§^	rn	0.32**266	
Non-malignant stroma PDGFRβ IR^§^			0.32**266
Gleason score^†^	rn	0.15**355	0.26**287
Local tumor stage^†^	rn	0.11*349	0.23**282
Tumor size^§^	rn	0.16**355	0.14*287
Tumor cell proliferation^§^(Ki-67 labeling index)	rn	0342	0.03282
Non-malignant epithelial cell proliferation^§^(K-i67 labeling index)	rn	0.22**348	0.12272
Tumor vascular density^§^(vWf stained vessels)	rn	0.10145	0.28**159
Non-malignant vascular density^§^(vWf stained vessels)	rn	−0.03144	0.02151

Notes: ^§^Pearson's correlation test and ^†^Spearman's correlation test. Data used in the correlation analysis were collected at the time of prostate cancer diagnosis.

*Correlation is significant at the <0.05 level (2-tailed).

**Correlation is significant at the <0.005 level (2-tailed).

Abbreviations: IR, immunoreactivity.

In some cases, the PDGFRβ expression also varied within the prostate tissue of the same patient (Figure S1).

### Stromal PDGFRβ expression occur predominantly in αSMA-positive cells

To further characterize the stromal PDGFRβ expression, a double staining was performed with antibodies against αSMA and PDGFRβ.

**Figure 2:**
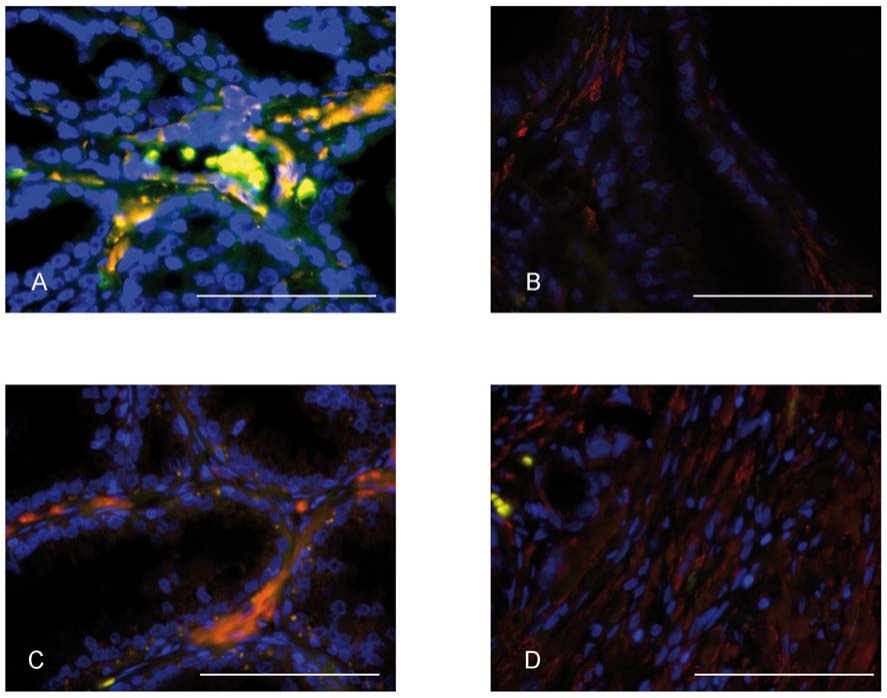
PDGFRβ is mainly expressed in αSMA-positive cells. Double stainings of prostate tissue with PDGFRβ (green) and αSMA (red) in non-malignant tissue (A and B) and tumor tissue (C and D). Scale bar = 100 µM.

These analyses confirmed that the majority of the PDGFRβ expression in the non-malignant fibromusclar and the fibroblast-like tumor stroma occurred in αSMA-positive cells (Figure 2). Furthermore, the analyses also demonstrated the presence of αSMA-positive cells in the stroma of tumor and non-malignant tissues which did not show any stromal PDGFRβ expression.

These findings thus confirm the existence of subsets of stromal αSMA-positive cells, which differ with regard to PDGFRβ expression.

### Stromal and perivascular PDGFRβ staining in prostate tumors is correlated with prognostic markers

Analyses were performed to investigate possible associations between PDGFRβ expression in tumors and clinical parameters or histological characteristics of the tumors.

**Figure 3:**
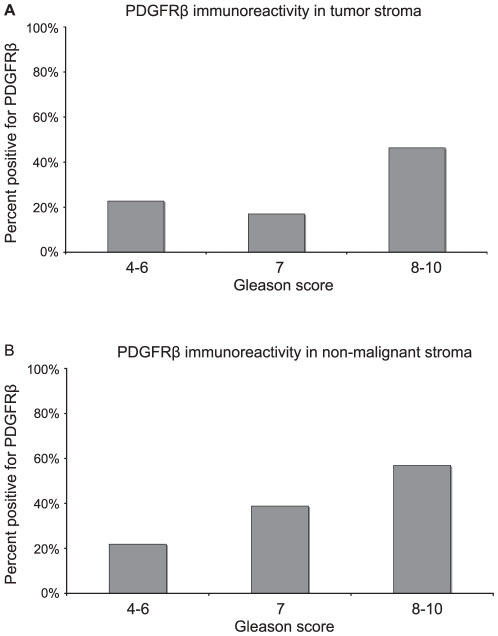
Stromal PDGFRβ expression is correlated with Gleason grade. PDGFRβ staining in tumor stroma was more common in cases with Gleason score 8–10 tumors as compared with patients with a Gleason score 4–6 or Gleason score 7 (A). Presence of PDGFRβ immunostaining was more common in the normal prostate tissue stroma when a Gleason score 8–10 tumor was present in the prostate compared to if the tumor had Gleason score 4–6 (B).

Presence of PDGFRβ staining in the fibroblast- like tumor stroma was significantly positively correlated with large tumor size, advanced stage, high Gleason score and high vessel density (Table 1). In patients with a Gleason score 4–6 or Gleason score 7 tumor, only about 20% of the tumors showed stroma PDGFRβ staining, but it was considerably more common in cases with Gleason score 8–10 tumors (Figure 3 A).

Perivascular PDGFRβ expression in the tumor area was also positively correlated with advanced stage, tumor vessel density and high Gleason score (data not shown).

Together these findings demonstrate previously un-recognized associations between stromal and perivascular PDGFRβ expression in prostate tumors and characteristics associated with a more aggressive cancer phenotype.

### Stromal PDGFRβ staining in the adjacent non-malignant tissue are correlated with prognostic markers

PDGFRβ expression in adjacent non-malignant tissue was also analyzed with regard to associations with tumor characteristics (Table 1).

High PDGFRβ in non-malignant, fibromuscular stroma was correlated with large tumor size, advanced stage, epithelial cell proliferation and high Gleason score (Table 1). It was considerably more common to find PDGFRβ expression in the normal prostate tissue stroma when a Gleason score 8–10 tumor was present elsewhere in the organ compared to if the tumor was Gleason score 4–6 (Figure 3 B).

Perivascular PDGFRβ expression in the surrounding non-malignant tissue was also positively correlated with epithelial cell proliferation (data not shown).

These analyses thus suggest novel relationships between prostate cancer properties and the phenotype of the stroma of the non-malignant prostate tissue adjacent to prostate tumors.

### Stromal PDGFRβ expression in tumors and in the non-malignant prostate tissue surrounding tumors predicts cancer specific survival

The results from the analyses of PDGFRβ expression were finally combined with survival data to investigate possible prognostic significance.

The cut-offs for tumor stromal PDGFRβ and non-malignant stromal PDGFRβ in the analyses was set to the third quartile, corresponding to 1.0 and 0.5 for tumour and non-malignant stromal PDGFRβ staining, i.e. high PDGFRβ immunoreactivity was ≥1.0 for tumour and ≥0.5 for non-malignant stroma.

**Figure 4:**
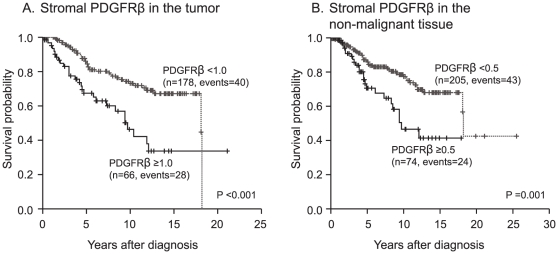
PDGFRβ expression in the stroma of tumors and non-malignant prostate tissue surrounding tumors predicts cancer specific survival. Patients divided into two groups depending on stromal expression of PDGFRβ in tumor (A) and non-malignant prostate tissue surrounding tumors (B). Solid line, high PDGFRβ in tumor stroma (≥1.0); dashed line, low PDGFRβ in tumor stroma (<1.0; A). Solid line, high PDGFRβ in non-malignant stroma (≥0.5); dashed line, low PDGFRβ in non-malignant stroma (<0.5; B).

Kaplan-Meier analysis showed that the watchful waiting patients with the highest quartile of PDGFRβ expression in tumor stroma had a significantly shorter cancer specific survival compared to the rest (15-year probability of event-free survival (P-EFS) was 67±5% and 34±9% in the two groups) (Figure 4 A).

Interestingly, high expression of PDGFRβ in the stroma of adjacent normal prostate tissue was also associated with a significantly reduced survival in patients managed by watchful waiting (15-year P-EFS was 68±5% and 41±9% in the two groups) (Figure 4 B).

High tumor and non-malignant stroma PDGFRβ immunostaining was associated with an increased relative risk for prostate cancer specific death in a univariate Cox regression analysis (Table 2). In multivariate Cox regression analysis including the known prognostic marker GS and local tumour stage, high stromal PDGFRβ in tumor and non-malignant tissue was not an independent prognostic marker (data not shown).

**Table 2:** Cox regression for stromal PDGFRβ in tumor and non-malignant tissue of patients followed with watchful waiting.

Variable		N	RR	*P*-value	95% CI
*Univariate analysis*					
Tumor stromal PDGFRβ**	<1.0	178	1*		
	≥1.0	66	2.4	<0.001	1.5–4.0
Non-malignant stromal PDGFRβ**	<0.5	205	1*		
	≥0.5	74	2.3	0.002	1.4–3.8

*Reference value.

**Cox regression analysis using stromal PDGFRβ as categorical variables.

Abbreviations: RR, relative risk; CI, confidence interval.

## Discussion

The present analyses uncovered a set of novel associations between the tumor stroma PDGFRβ status and histopathological characteristics, including positive correlations with Gleason score, tumor stage and tumor size (Table 1). These findings are reminiscent of the recently described situation in breast cancer where stromal PDGFRβ expression was positively correlated with high grade [21]. These findings raise the question of the underlying mechanism(s). As of now it is not possible to conclude whether the associations are caused by an epithelial-induced stromal phenotype or by a stroma-induced epithelial phenotype.

The analyses of the present study also revealed significant associations between clinical characteristics, including survival, and the stromal PDGFRβ expression in the non-malignant present tissue (Table 1). This observation of prognostically significant properties of the non-malignant tissue adds to a series of similar observations that together have led to proposing the term “tumor indicating non-malignant tissue” (TINT), which implies that analyses of non-malignant prostate tissue can yield prognostic information [2], [10]. Other recent studies have presented similar findings following analyses of e.g. pAKT, pEGFR and AR [2], [10], [22]. From a practical perspective this is important since many diagnostic biopsies only sample non-malignant tissue.

The underlying biology of the variations in stromal PDGFRβ expression should be better clarified. From the present analyses it is unclear whether the alterations in the non-malignant tissue are secondary to the presence of near-by malignant tissue as suggested in animal models [8], or if it rather reflects a tumor-permissive environment which causally contributes to formation of more malignant tumors.

The analyses including double staining with αSMA and PDGFRβ antibodies revealed that the PDGFRβ expression occurred in αSMA -positive cells (Figure 2). However, it was also noted that these cells, in tumors scored as having a PDGFRβ-positive stroma, occurred together with αSMA -positive/PDGFRβ-negative cells. These findings thus clearly exemplify the molecular heterogeneity among the cells of the non-malignant fibromusclar stroma and of the fibroblast-like tumor stroma. Similar findings have been made in experimental tumors [23]. As also suggested by others [24], the findings should stimulate to an improved classification of these subsets and continued analyses of their functional significance.

The associations between high stromal PDGFRβ expression and shorter survival indicate that this receptor is causally linked to the clinical course of the disease and thus also suggests targeting of PDGF receptors for therapeutic purposes. Some smaller studies have been reported where imatinib have been used in prostate cancer without any obvious effects [25], [26]. The findings from the present study, demonstrating large variations in stromal PDGFRβ expression, suggest that future studies evaluating this approach will require more stringent procedures for patient selection. In this context it can also be noted that epithelial PDGFRβ expression was only rarely detected in this study (data not shown). Furthermore, no associations between this expression and clinical or histopathological characteristics were observed.

A series of topics for future studies are suggested by the present findings. Experimental studies involving co-cultures of epithelial and stromal cells should help to clarify if the stroma-epithelial associations have their predominant mechanistic basis in the stromal or epithelial compartment. Another interesting topic for future studies would be to investigate to what extent the stromal and epithelial phenotypes are linked to individual prostate cancer risk genes. Finally, continued analyses are required to understand if the tumor-associated variations in the non-malignant tissue are a cause or a consequence of the tumor formation. Careful tissue analyses in prospective studies might be required to clarify this issue.

## Materials and Methods

### Patients and tissue microarray

Tissue specimens were collected from patients who underwent transurethral resection of the prostate (TURP) at the hospital in Västerås, Sweden, between 1975 and 1991. Histological analysis showed presence of prostate cancer. Median age at TURP was 74 years (range 51–95 years). Information concerning absence or presence of benign prostate hyperplasia was not available. Tissue specimens were formalin-fixed and paraffin-embedded followed by regrading according to the Gleason system. The specimens were used to construct a tissue micro array (TMA) using a Beecher Instrument (Sun Prairie, WI, USA). The TMA:s contained 5–8 samples of tumor tissue representing both the primary and secondary Gleason grade and 4 samples of non-malignant tissue from each patient. The patients had not received any anti-cancer therapy before TURP. Radio nuclide bone scan was performed shortly following diagnosis for detection of metastases. There were 377 patients included in the study, of which 293 patients were followed with watchful waiting after TURP. At symptoms from metastases patients received palliative treatment with androgen ablation and in a few cases radiation therapy or oestrogen therapy, according to therapy traditions in Sweden during that time. Also, 84 patients that were treated with palliative treatment immediately after diagnosis were analyzed. The median overall survival for the patient group followed with watchful waiting was 5.6 years. Ninety-two of the TURP specimens were graded as Gleason score (GS) 4–5, 107 patients as GS 6, 63 patients had GS 7, and 115 patients GS 8–10. 3 patients (4.1%) with GS 6, 4 patients (8.2%) with GS 7, and 33 patients (33.7%) with GS 8–10 had bone metastases at diagnosis. In August 2003, 36 patients (9.5%) were still alive, 131 patients (34.7%) had died from prostate cancer and 210 patients (55.7%) had died from other causes. The material was collected according to Swedish regulations at a time when informed consent was not required. The research ethical committee at Umeå university hospital (Regional Ethical Review Board in Umeå) approved of the study and waived the need for consent.

In this material we have already analyzed factors of potential prognostic significance such as Gleason score, tumor volume, tumour stage, tumor cell proliferation and vascular density and the data obtained were now related to the current PDGFRβ findings [2], [27].

### PDGFRβ immunohistochemistry

PDGFRβ immunohistochemistry was performed as described in Nupponen *et al* 2008 [28]. Anti- PDGFRβ (rabbit monoclonal, #3169, Cell Signaling Technology, Danvers, MA, USA) was used at a concentration of 2 µg/ml. The staining intensity was scored separately in stroma and around vessels as negative (0), weak (1), moderate (2) or strong (3). The PDGFRβ staining score are the median values of five to eight scored samples of tumor tissue or four scored samples of nonmalignant tissue. For correlation analysis the samples were scored as positive for PDGFRβ if staining was detected in at least one of the TMA cores.

### Immunofluorescence

Immunofluorescent stainings of the TMAs was performed as above (in the immunohistochemistry section) until the incubation with the anti- PDGFRβ antibody. Sections were then washed in PBS with 0.1% Tween-20 (PBT) 3×5 min. This was followed by a 1 h incubation with the second primary antibody, mouse monoclonal αSMA (M0851, clone 1A4, Dako Cytomation, Glostrup, Denmark) diluted 1∶100 in 20% goat serum in PBT. After washing in PBT for 3×5 min, slides were incubated with biotinylated goat anti-mouse antibody (E0432, Dako Cytomations 1∶500) for 45 min at RT. This was followed by another 3×5 min washing in PBT. The slides were then incubated with Alexa-Flour 488 goat anti-rabbit secondary antibody (A11008, Invitrogen, Carlsbad, CA, USA) diluted 1∶100 in PBT for 45 min at RT. Following washing; the slides were incubated for 45 min with Cy3-Streptavidin conjugate (Sigma-Aldrich) diluted 1∶500 in PBT for 45 min at RT. After washing the slides were dried and mounted with Vectashield mounting medium with DAPI (Vector Laboratories, Burlingame, CA, USA).

### Statistics

Bivariate correlations were calculated with the Pearson's correlation test. Correlations between categorical variables and continuous variables were analyzed using the Spearman's rank correlation test. Data was collected at the time of prostate cancer diagnosis.

Patients included in survival analyses with the Kaplan-Meier and Cox regression were followed with watchful waiting. The duration of event-free survival (EFS) is defined as the time from TURP until the date of prostate cancer death, death of other cause, or until the date of last follow-up. Event in the survival analysis was defined as prostate cancer death, thereby showing cancer-specific survival. Differences in outcome were tested with the log-rank test. The prognostic significance of PDGFRβ immunoreactivity was evaluated with Cox regression analysis alone and combined with GS and local tumour stage. Probability of event-free survival (P-EFS) is presented±standard error (SE). The level of statistical significance was defined as *P* <0.05 (two-sided). Statistical analysis was performed using the SPSS 17.0.0 software for OS X (SPSS Inc., Chicago, IL, USA).

## Supporting Information

Figure S1 PDGFRβ expression occasionally varies within the prostate tissue of the same patient. Heterogenous PDGFRβ staining patterns was observed in many tumors. A and B illustrate different parts of the same prostate cancer. Scale bar = 200 µM.
(7.24 MB EPS)
